# Genome-Wide DNA Methylation Patterns and Transcription Analysis in Sheep Muscle

**DOI:** 10.1371/journal.pone.0101853

**Published:** 2014-07-10

**Authors:** Christine Couldrey, Rudiger Brauning, Jeremy Bracegirdle, Paul Maclean, Harold V. Henderson, John C. McEwan

**Affiliations:** 1 AgResearch Ltd., Ruakura Research Centre, Hamilton, New Zealand; 2 AgResearch Ltd., Invermay Agricultural Centre, Puddle Alley, Mosgiel, New Zealand; 3 AgResearch Ltd., Grasslands Research Centre, Palmerston North, New Zealand; Institute of Farm Animal Genetics, Germany

## Abstract

DNA methylation plays a central role in regulating many aspects of growth and development in mammals through regulating gene expression. The development of next generation sequencing technologies have paved the way for genome-wide, high resolution analysis of DNA methylation landscapes using methodology known as reduced representation bisulfite sequencing (RRBS). While RRBS has proven to be effective in understanding DNA methylation landscapes in humans, mice, and rats, to date, few studies have utilised this powerful method for investigating DNA methylation in agricultural animals. Here we describe the utilisation of RRBS to investigate DNA methylation in sheep *Longissimus dorsi* muscles. RRBS analysis of ∼1% of the genome from *Longissimus dorsi* muscles provided data of suitably high precision and accuracy for DNA methylation analysis, at all levels of resolution from genome-wide to individual nucleotides. Combining RRBS data with mRNAseq data allowed the sheep *Longissimus dorsi* muscle methylome to be compared with methylomes from other species. While some species differences were identified, many similarities were observed between DNA methylation patterns in sheep and other more commonly studied species. The RRBS data presented here highlights the complexity of epigenetic regulation of genes. However, the similarities observed across species are promising, in that knowledge gained from epigenetic studies in human and mice may be applied, with caution, to agricultural species. The ability to accurately measure DNA methylation in agricultural animals will contribute an additional layer of information to the genetic analyses currently being used to maximise production gains in these species.

## Introduction

The rapid advances that have been made in DNA sequencing technologies over the past 20 years [1] have resulted in genome-wide selection for production traits becoming a reality in agricultural animals [2]. This is allowing the animal breeding industry to significantly increase the rate of genetic progress. This technology will allow all sequence variations in each individual in closed breeding schemes to be known. What is not currently known is how to rank these variations, especially those that modify gene expression rather than amino acid sequence. In order to continue making genetic progress we will need, among other things, an understanding of how epigenetics affects gene expression and ultimately phenotype.

Epigenetics is the study of changes in phenotype caused by mechanisms other than changes in the underlying DNA sequence. Epigenetic mechanisms include, but are not limited to: DNA methylation, acetylation and methylation of histone proteins that bind and stabilise DNA, and non-coding RNA molecules. These epigenetic processes act in an interrelated manner in order to regulate gene expression. One of the key determinants in the control of gene expression in mammals, and the most common covalent modification of DNA in eukaryotes, is cytosine methylation at CpG dinucleotides. Abundant evidence indicates that CpG methylation plays a central role in many aspects of biology including growth, development, genomic imprinting, and X-chromosome inactivation in females (reviewed by [3]). Similarly aberrant DNA methylation can preclude normal development and lead to disease (reviewed by [4]). Despite the clear importance of DNA methylation, it remains poorly understood how methylation patterns are set during development, maintained, change in response to environmental conditions, regulate gene expression, and in some cases how they affect gene expression in offspring.

Massively parallel DNA sequencing has recently become a vital tool in the analysis of DNA methylation and RRBS has proven to be effective in understanding DNA methylation landscapes ([5] [6] [7]). However, to date, mammalian genome-wide epigenetic studies have focused on humans and mice. Here we describe the use of a RRBS protocol optimised for sheep, in order to offer a system for identifying (epi)mutations that correlate with economically important agricultural phenotypes across the whole genome. *Longissimus dorsi* (LD) muscle was chosen from animals of known wildtype genotype status at a locus known to affect muscle dimension, inherited in a parent of origin fashion. This study of LD muscle methylome is a first step analysing the potential epigenetic involvement in the muscle hypertrophy phenotype study from which these wildtype animals originated. However, the study will also form the basis of understanding the involvement of epigenetic regulation of muscle growth which, in terms of production performance, especially in the high value muscles, is the single most important trait in New Zealand sheep. Furthermore, many of the factors affecting the eating qualities of meat (tenderness, juiciness, odour and flavour) are environmental in nature and therefore epigenetic modifications are likely to play an important role in the final eating quality. Together these factors make the LD muscle and exemplar tissue in which to begin examining the role of epigenetics in sheep.

## Materials and Methods

LD samples from three wild-type eight month old Poll Dorset lambs were collected at a research abattoir (AgResearch Invermay) under Invermay Animal ethics committee tissue collection application number 11. A 1 cm sliver was cut from the middle of the muscle to ensure consistency across samples. Muscle samples were snap frozen in liquid nitrogen within 15 minutes of slaughter and stored at −80°C until processed. High quality DNA and RNA were extracted [8]. RRBS methodology based on previously published RRBS studies ([5] [6] [7]) and modified for use with sheep samples [9] was used to quantify DNA methylation levels across the genome. Briefly MspI restriction enzyme was used to digest 5 µg genomic DNA at 37°C overnight. The extent of digestion was checked by electrophoresis on a 1% agarose and the remainder of the digestion was cleaned using DNA Clean and concentrator-25 columns (Zymo, CA, USA). Sticky ends produced by MspI digestion were filled with CG nucleotides and Illumina sequencing adapters (Illumina, CA, USA) containing methylated cytosines were ligated onto digested DNA following the manufacturer’s protocols (Illumina TruSeq library preparation kit). Ligation reactions were purified using DNA Clean and concentrator concentrator-5 columns (Zymo, CA, USA).

Size selection was performed manually on a 3% nusieve agarose gel (Alphatech, NZ) to capture insert sizes of 150–250 bp based on previous studies (Doherty and Couldrey 2014). Efficiency of adaptor ligation and size selection was determined by qualitative PCR as previously described [9].

Bisulfite conversion of non-methylated cytosines was performed on size-selected fragments using an EZ-DNA bisulfite conversion kit (Zymo CA, USA) following the manufacturer’s instructions, except for a modification to bisulfite conversion conditions as recommended by Smith et al. 2009: 99°C for 5 min, 60°C 25 min, 99°C 5 min, 60°C 85 min, 99°C 5 min, 60°C 175 min, 6×(95°C 5 min, 60°C 90 min). Small scale test PCR amplification using primers in Illumina TruSeq kit was performed on converted DNA using 15, 20, 25 PCR cycles to determine the minimum amount of amplification to be performed (Doherty and Could rey 2014). The remainding 20 µl of bisulfite treated DNA was amplified for 15–20 PCR cycles in four 100 µl reaction volumes. PCR reactions for were purified using Clean and concentrator -5 column (Zymo CA, USA), analyzed on a bioanalyzer (Agilent, CA, USA) and each library was sequenced on one lane of an Illumina HiSeq sequencer using 100 bp paired-end reads (National Centre for Genome Resources, Santa Fe, NM). RRBS was performed in duplicate on one sample to determine the repeatability of the methodology. Quality control of data was undertaken using FastQC software (Babraham bioinformatics, UK). The first two nucleotides from all read_2 sequences were trimmed as these were uninformative and resulted from the blunting of MspI site. BWA style trimming [10] was performed on all reads using a Phred quality score of 20 as the minimum value. Sequences were mapped using paired end mapping to the sheep genome assembly OARv3.1 using Bismark software (Babraham bioinformatics, UK). Following optimisation, a seed length of 50 bp was chosen and only one mismatch was tolerated. Only sequences in which both ends mapped uniquely in the correct orientation with an appropriate insert size (150–250 bp) were used for subsequent calculation of DNA methylation levels.

Sequencing read counts and levels of methylation were calculated and visualised using Seqmonk software (Babraham bioinformatics). Genome-wide DNA methylation analysis was performed at three different levels of resolution: average methylation across and between annotated genes, average methylation around transcription start sites (TSS) using a 1000 bp sliding window with a step size of 50 bp, and single nucleotide resolution. Where necessary, average DNA methylation levels were extracted from Seqmonk data files and CpG content surrounding TSS was calculated using Python scripts developed in-house.

Accuracy of RRBS protocol was assessed by comparing DNA methylation levels at 134 selected CpG sites on chromosome 18 (at locus known to alter muscle growth) measured by Sequenom MassARRAY (Sequenom, CA, US) on the same three LD samples using protocols previously described [11], [12]. Primer sequences for Sequenom assays are listed in table 1 with each primer also containing the standard Sequenom tags, forward AGGAAGAGAG, and reverse CAGTAATACGACTCACTATAGGGAGAAGGCT.

**Table 1:** Sequenom PCR primer sequences used to determine DNA methylation on chromosome 18.

Assay	Left primer	Right primer	Strand
Ch18_1	GGTTTGAAATATGGAGGGTTAGTTA	CAACCAACAAATACATACCAAATCT	forward
Ch18_2	TGGTTAGTGAGGTTTTAGATTTGGT	ACCCAATTCACAAATACAAAAAAAA	forward
Ch18_3	TTTTTTTTGTATTTGTGAATTGGGT	TCAAAACTAAACCACCAAAACTACC	forward
Ch18_4	AGGTTTGATAGAAGGTGGTAAATGA	ACTTAAACCCACAAACTCATACCAA	forward
Ch18_5	GTTATGAGGTTTATTATTTTGGGGG	TAATAACCATCTCAAAAACCCTTCA	forward
Ch18_6	TGAAGGGTTTTTGAGATGGTTATTA	TTACTAAACCCATACCCCACCTAAC	forward
Ch18_7	GGGAGTTTTATAGGAGTTAGGAGTAG	CCCTACAAAACAAAACTTAAAACCA	reverse
Ch18_8	GAATTTTGGAGTGGGTAGTTTTTTT	CTTACACTAAACCCTCCTTCCAAAT	forward
Ch18_9	TTGATGATGGTTTGGAAGTAGTGTA	CTCAAAAAATACCTTAAACCCCACT	forward
Ch18_10	TGGGGTTTAAGGTATTTTTTGAGAT	CAAAACCCAAAACTCCTCACTAATA	forward
Ch18_11	GATTGAATTGAGTTGAATTGGGTT	TCAAACAAAAACCTCAACCATAAAT	forward
Ch18_12	TTTTAGTTTAATGTTGGGTATGTGG	CCTAAACAAAAAAAACCAAAACAAA	forward
Ch18_13	GGGTGTTAGTGGAGTAGATGAGTATTT	ACAAACAACCTAACAACCAAAAAAA	forward
Ch18_14	TTTGTGGAGTGTTTATTGTGTGTAGA	CCCTAAACTAAAAACTCACCCTCTC	forward
Ch18_15	GGTTTTATAGAGGAGATTGTGTTGTTTT	CACAACCCTTACTACAAATAAAACCT	forward
Ch18_16	GTTTTTGTTTTTTGTTTGGGTAGTG	AATCCAAAAAAACCTTCCTACAAAA	forward

Next generation sequencing of LD mRNA was performed by Macrogen (Korea). Data was downloaded and regions of low quality sequence, primers and adapters remaining from the sequencing process were removed from the reads prior to mapping to the sheep genome (OARv3.1) in order to determine gene expression levels.

### Statistics

Paired t-test analysis was used to determine whether calculation of DNA methylation levels by RRBS and Sequenom analysis were significantly different. Paired t-tests across three animals were used to determine whether average methylation levels around TSS were different between genes displaying high levels of expression and those genes that were repressed.

## Results

### Sequencing, quality control and mapping

The ovine LD muscle DNA was used for RRBS to generate data suitable for analysing DNA methylation from a genome-wide perspective as well as at the level of single nucleotide resolution in sheep. Illumina HiSeq 2000 sequencing generated 60–110 million high quality sequence reads for each of the biological and technical replicates (accession number PRJNA248306). Quality control analysis using FastQC indicated that the 100 bp sequences displayed the expected nucleotide composition based on MspI digestion and bisulfite conversion. On average, 97% of read_1 sequences began with CGG or TCC with the remaining read_1 sequence being very C-poor and T-rich together with 97% of read_2 sequences starting with CAA and T-rich and being G-poor and A-rich as previously described [9].

Sequences were mapped to OARv3.1 using Bismark software. Mapping parameters were optimised for the sheep genome and capacity of servers currently available for analysis. Genome-wide DNA methylation was able to be determined at single nucleotide resolution in regions selected by reduced representation. For each sample 58–62% of sequences mapped uniquely to the genome. Sequences that did not map, or did not map uniquely were not included in the analysis. Non-CpG methylation was less than 1% for each sample indicating high sodium bisulfite conversion efficiency.

Repeatability of the RRBS protocol at single nucleotide level was determined by comparison of technical replicates. Depending on the stringency of analysis, the correlation of RRBS technical replicates varied between r = 0.82, when the minimum number of reads covering a CpG site was set at 10 (which is also the default setting in Seqmonk), to r = 0.95 when a minimum of 100 reads were required for a CpG site to be included in the analysis.

**Figure 1:**
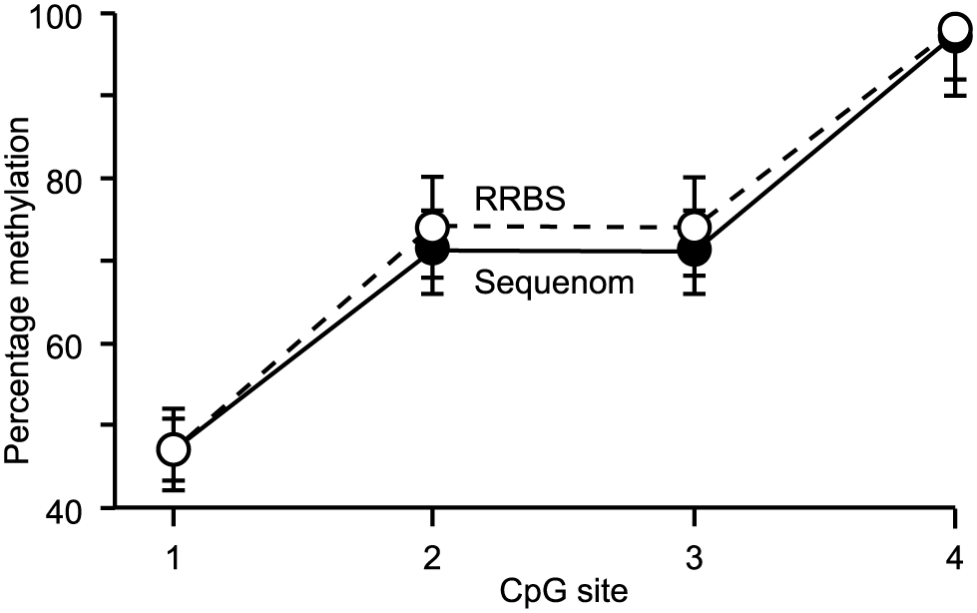
Accuracy of RRBS. Comparison of DNA methylation levels at single nucleotide resolution obtained using RRBS and levels measured at the same sites using Sequenom analysis. Four neighbouring CpG sites are shown as an example of concordant results.

A comparison of data from the RRBS methylation and Sequenom analysis (a technique that has proven useful in analysis of DNA methylation in the past [11], [13] of 134 CpG sites selected on chromosome 18 identified that the percentage methylation measured by the two independent methods were indistinguishable at 132/134 CpG sites. An example of this accuracy is illustrated in Figure 1.

### Genome-wide DNA methylation

**Figure 2:**
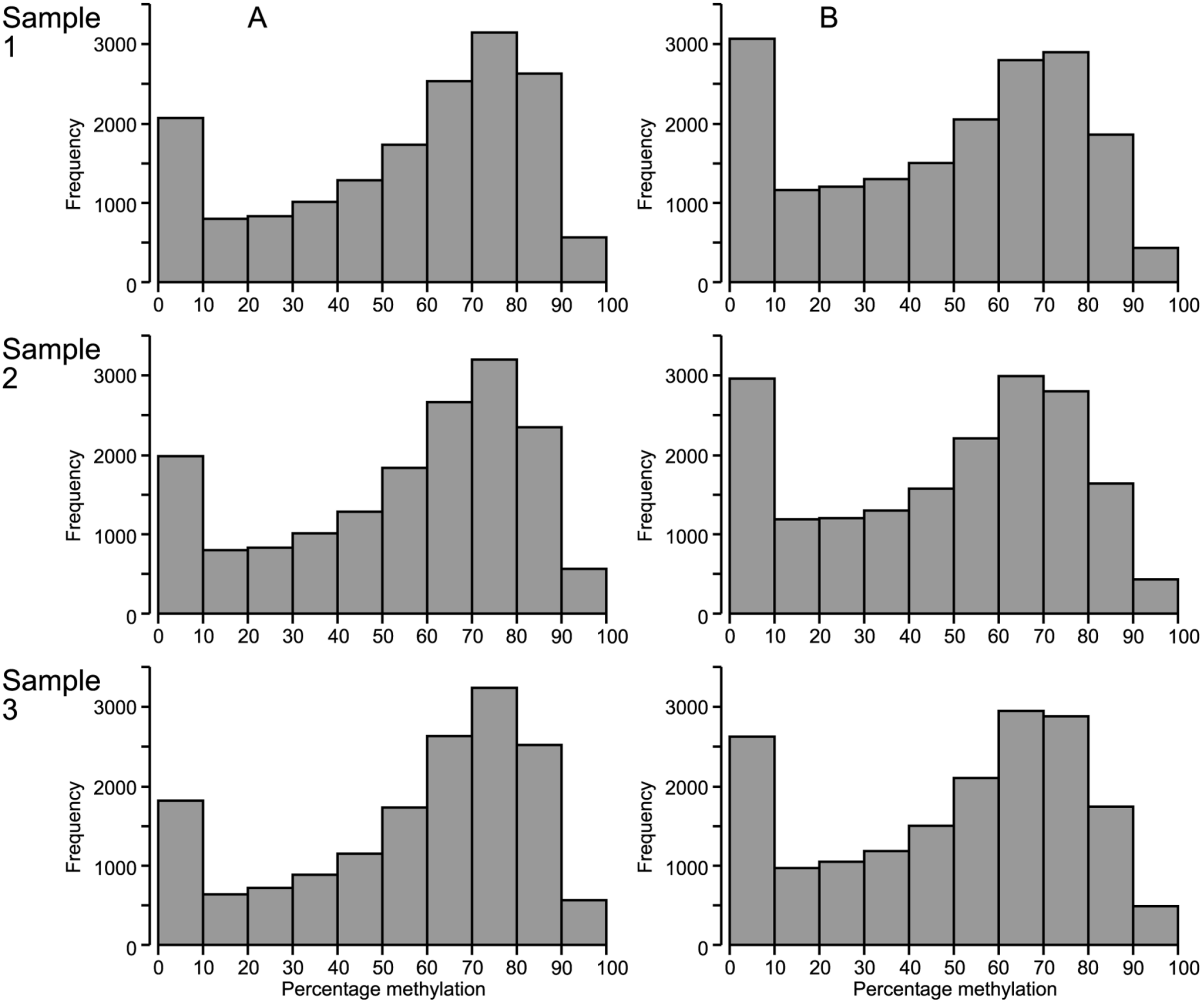
Average DNA methylation in regions annotated as genes and intergenic regions. Histogram of average DNA methylation calculated for (a) annotated genes and (b) intergenic regions.

Sequencing and mapping data indicated that 50–55% of CpG sites included in RRBS analysis were methylated. Analysis of average DNA methylation in regions annotated as genes showed a bimodal distribution, with peaks at 0–10% methylation and 60–80% methylation (Figure 2a). Average methylation across intergenic regions showed a similar distribution to that seen in annotated genes (Figure 2b).

### Methylation and CpG content around transcription start sites (TSS)

**Figure 3:**
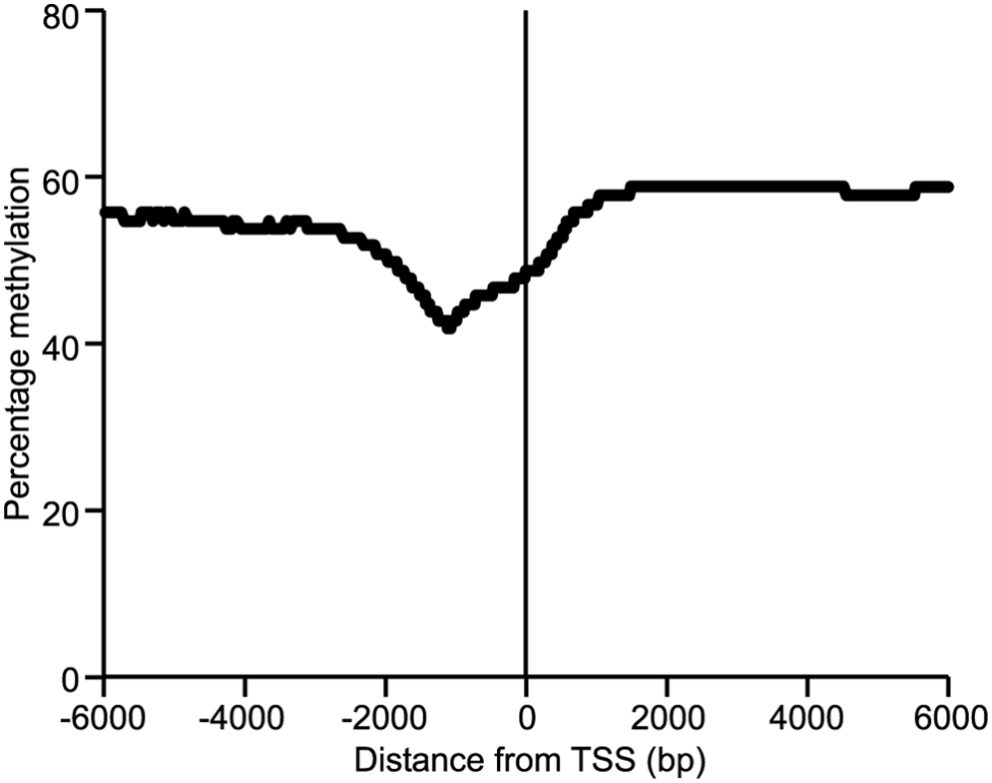
DNA methylation around transcription start sites. Average DNA methylation 6% for each data point and therefore too small to be represented in the figure.

**Figure 4:**
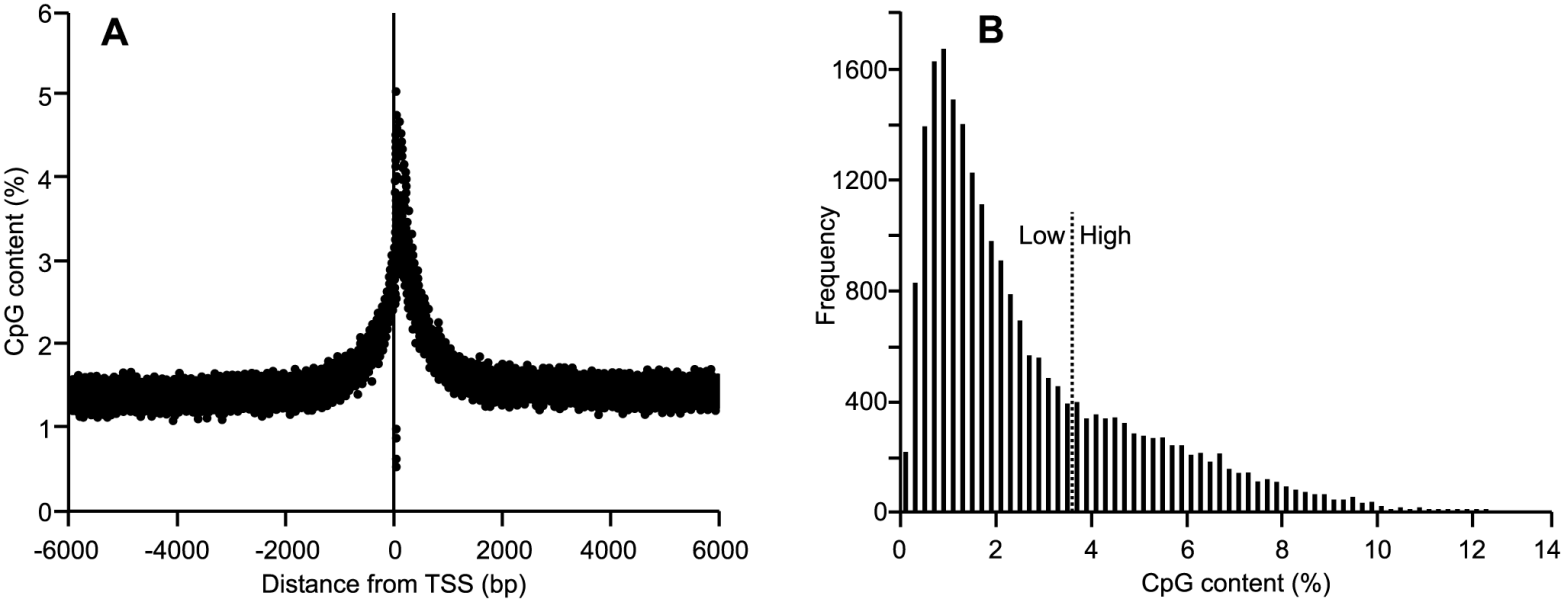
CpG content around transcription start sites in the sheep genome (OARv3.1). CpG content 6(A), and a histogram of gene counts with 0–14% CpG content in 1000 bp around the TSS (B).

Average DNA methylation 6 kb upstream and 6 kb downstream from all annotated genes in the sheep genome was calculated using a sliding window approach with 1000 bp window size and 50 bp step size. On average the DNA sequence directly upstream of the TSS showed less methylation than surrounding sequence (Figure 3). *In silico* analysis of CpG content across the same regions identified a peak of CpG content at the TSS (Figure 4a). However, when a 1000 bp region surrounding the TSS was examined on a gene by gene basis, it was found that although some genes had as high as 10–13% CpG content surrounding the TSS, many genes had as little as 1 or 2% (Figure 4b). Genes were designated “high CpG content” or “low CpG content” depending on whether they had greater than or less than 3.7% CpG content within the 1000 bp surrounding the TSS. This division was based on previously published work in rat [14].

### Methylation around TSS relative to gene expression and DNA sequence surrounding TSS

**Figure 5:**
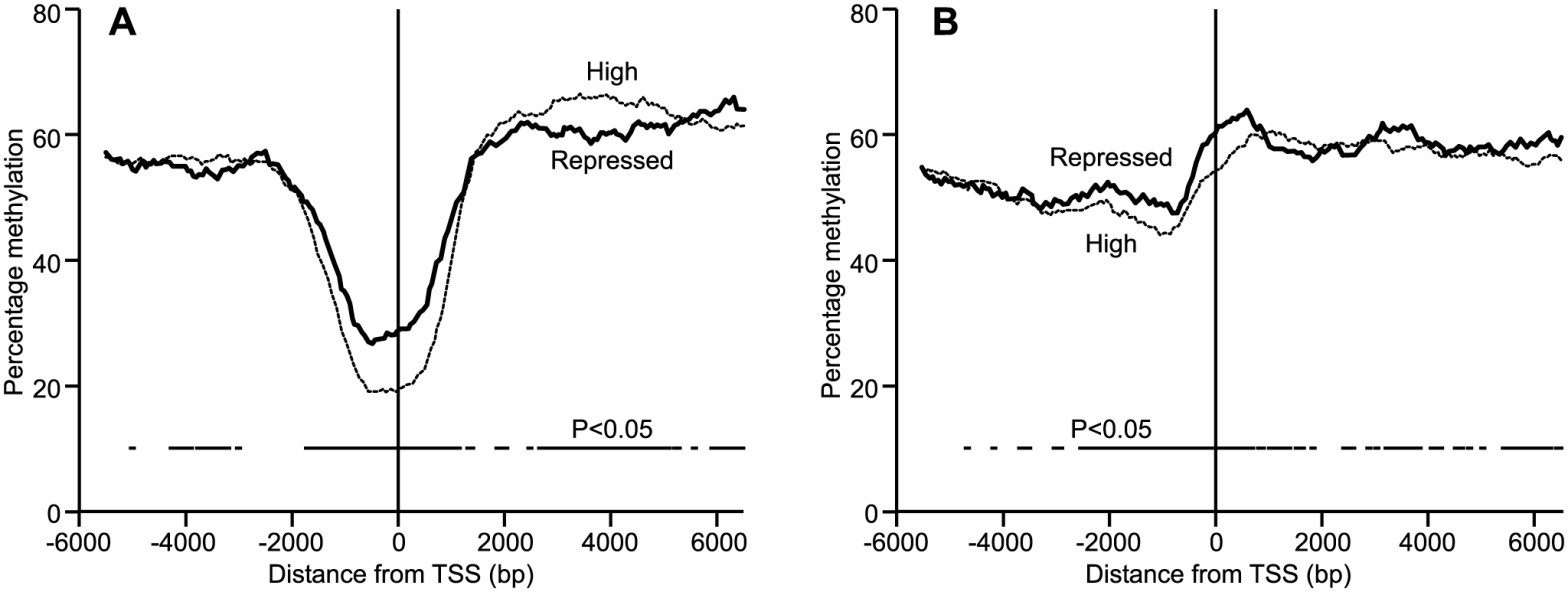
DNA methylation around transcription start sites relative to CpG content and gene expression. Average DNA methylation of highly expressed vs. repressed genes in three sheep across 6; a) genes with high CpG content, b) low CpG content. Standard error of the difference was less than 1.3% for each data point and therefore too small to be represented in the figure.

RNA sequencing resulted in 93–106 million reads per sample. Mapping efficiency was 82% for each sample. Genes were divided into three groups based on their expression levels determined by transcriptome sequencing: repressed genes represented by fewer than 10 reads (60% of all genes), low expression (20% of genes), and highly expressed (20% of genes). Based on these groupings, average DNA methylation around the TSS indicated that genes that were highly expressed had, on average, lower levels of DNA methylation immediately upstream of the TSS in genes with high CpG content (Figure 5a). Genes with low CpG content around the TSS showed similar, but less dramatic, methylation differences upstream but not downstream of TSS (Figure 5b).

### Single nucleotide resolution

**Figure 6:**
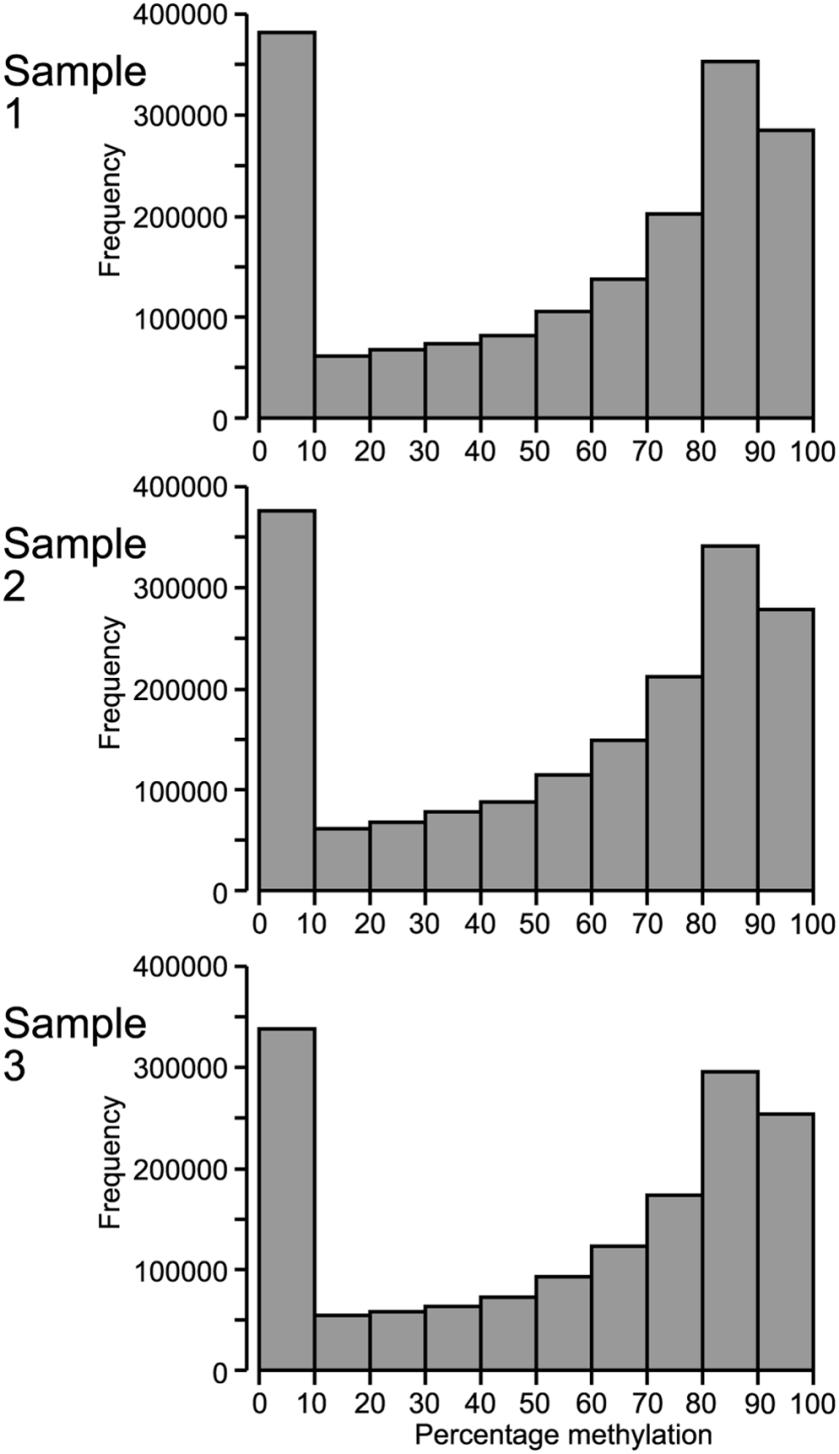
Single nucleotide resolution analysis of DNA methylation. Histogram of average DNA methylation calculated for all single CpG sites with a minimum read depth coverage of 10.

Across the three samples analysed, a total of 2,495,309 cytosines had a sequence coverage depth of at least 10 (minimum coverage for accurate and precise calculation of DNA methylation at single nucleotide level). Of these sites, 1,732,013 were able to be analysed in all three samples. Percentage DNA methylation was calculated at all CpG sites with ≥10X coverage. An example of the frequency distribution of DNA methylation at single nucleotide resolution across the genome in one sample is displayed in Figure 6. A bimodal distribution is seen, similar to that observed in DNA methylation analysis at the gene level (Figure 2), although the second peak is located at a higher level of methylation (Figure 6).

## Discussion

Fragmentation of the genome with restriction enzyme MspI and construction of directional RRBS libraries resulted in high quality sequencing libraries that were generated from approximately 1% of the genome. Sequencing of these libraries resulted in ∼7% of CpG sites in each sample having sufficient coverage depth to allow single nucleotide analysis (accurate calculation is challenging due to the presence of repeat regions in the genome). This level of enrichment of CpG sequencing suggests a dramatic cost saving compared to sequencing the entire genome following bisulfite conversion.

The high concordance of DNA methylation levels measured by RRBS and Sequenom analysis indicates that the RRBS technique is able to provide an accurate assessment of DNA methylation at the single nucleotide level. Similarly the good correlation of methylation levels measured in technical replicates and the minimal variation observed between biological replicates indicates that this method is not only accurate, but also precise. The epigenomic methodology used in this study is therefore sufficient to allow high resolution analysis for the comparison of different phenotypes. Taken together, these quality control parameters indicate that the RRBS library construction process resulted in high quality libraries.

The global CpG methylation of 50–55% measured by RRBS in these experiments is lower than the 80% methylation traditionally reported in somatic tissues (80%) using techniques such as high performance liquid chromatography (HPLC) only able to determine total methyl content [15]. This discrepancy is likely due to the requirement of unique mapping to the sheep genome when next generation sequencing methods are used to interrogate DNA methylation, and/or the predominance of MspI sites in promoter regions that often display low levels of DNA methylation [9], [16].

Care must be taken when drawing conclusions from epigenomic analysis, as bimodal distribution of DNA methylation was observed regardless of analysis chosen: DNA methylation analysed as average gene methylation, average methylation across intergenic regions, or methylation at the single nucleotide level. The ease at which large quantities of data are able to be generated now often necessitates visualising average values across the genome. While such visualisation can be useful, there is a high risk of losing useful and meaningful data in the process. This risk highlights the need for biologists’ involvement in bioinformatics analysis to ensure useful and specific questions are asked of the data.

Given the lack of resolution afforded by analysing the average DNA methylation across genes, a more in depth analysis of annotated TSS was conducted as DNA methylation levels around TSS are thought to be involved in regulation of gene expression [17]. Findings in this study that indicate lower levels of DNA methylation around TSS correspond well with what has been reported in other species [14]. However, to better understand the potential involvement of DNA methylation and transcription in sheep muscle it was necessary to undertake RNA-Seq on the same samples for which RRBS data was generated. This allowed us to distinguish gene expression levels and correlate this with DNA methylation around TSS.

Since the early days of investigating DNA methylation involvement in gene regulation, the common consensus is that less methylated DNA is likely to be in a more relaxed state and therefore the gene more open to transcription [18]; the results presented here support this dogma. However, the difference in methylation levels seen at TSS, while significant, was not as great as would be expected if this were the sole regulator, thereby suggesting that other factors are also involved. One such factor may be the DNA sequence around the TSS i.e. the effect of promoters and enhancers binding to the TSS and nearby regions.

Analysis of 12 kb surrounding the TSS revealed that CpG content was highest at the TSS. This high CpG content corresponds to presence of CpG dense “CpG islands” known to reside at the start of many genes [19]. Further analysis of CpG content for each individual gene 500 bp upstream to 500 bp downstream of the TSS revealed that the CpG content at the TSS varied from 0 to 13.4%. The frequency distribution of CpG content at TSS peaks at 1.2% and does not show the bimodal distribution seen in other species [14] including cattle (Figure S1). It is not clear from this data, whether this difference represents a true species difference, is tissue specific, or rather reflects the state of completion of the reference genome, as it is known that the current assembly of the sheep genome (OARv3.1) is lacking in sequence around the start of ∼10% of annotated genes (personal communication: International Sheep Genomics Consortium), a region that typically contains higher than average levels of CpG dinucleotides. Because no bimodal distribution was seen in the frequency of TSS CpG content, genes were divided into “high” or “low” CpG content based on the distribution seen in other species with the split made at 3.7%.

Analysis of DNA methylation levels around the TSS based on CpG content and expression level illustrates that methylation is significantly different between highly expressed and repressed genes across much of the 12 kb region spanning the TSS analysed regardless of CpG content at TSS. At the same time the levels of DNA methylation around TSS are dramatically different between genes with high and low CpG content at TSS. Furthermore, differences in DNA methylation between highly expressed and repressed genes downstream of TSS depend on CpG content around the TSS. This latter point is notable in that there is a growing body of evidence in other species (such as humans and rats) that DNA methylation in the first exons and introns of genes is correlated with gene expression [20]–[22]. Therefore, our data suggests that some epigenetic control mechanisms in sheep are likely to be similar to those in other mammalian species.

While performing a sliding window approach for DNA methylation analysis in sheep was able to provide an interesting overview of TSS analysis, the true power of the RRBS approach to DNA methylation analysis is its ability to provide single nucleotide resolution of DNA methylation. The bimodal distribution of DNA methylation at single nucleotide level showed that the majority of sites in sheep LD muscle are at the extremes, either always unmethylated or always methylated, with a much smaller proportion of sites found to be methylated in some cells but not others or on one but not both parental chromosomes within individual cells. Although this data fits well with the traditional notion of DNA methylation whereby sites have been classified as methylated or unmethylated, direct comparison of these data with other species is not possible at this time due to a lack of studies that have a) sequenced samples deeply enough to allow single nucleotide resolution, b) sequenced LD muscle, and c) sequenced the same heterogeneous cell pool of multinucleate muscle fibres and quiescent muscle satellite cells as undertaken in the present study. The cellular heterogeneity is a major challenge in comparing DNA methylation across tissue samples and species. However, in future such high resolution analysis of DNA methylation across the genome will provide a powerful tool for identifying epigenetic modifications that are able to contribute to the phenotypes of agricultural animals.

Taken together, although optimisation of RRBS was required for successful analysis of DNA methylation in sheep, this work has identified many similarities in genome-wide DNA methylation patterns between sheep and other animals. The similarities between sheep and species more commonly used for epigenetic studies, such as humans and rats, is promising in that knowledge gained in these species can be transferred to agricultural animals. This work also highlights the complexity of epigenetic regulation of genes. It appears unlikely that regulation of gene expression will be entirely controlled by a particular epigenetic process; rather, it is likely that a combination of epigenetic processes, together with the underlying genomic sequence will interact synergistically to ensure appropriate growth and development. As our understanding of DNA methylation and other epigenetic mechanisms increases, the future of improving production performance in agricultural animals will likely involve not only genetic selection but also epigenetic information to maximise production gains in these species.

## Supporting Information

Figure S1 **Transcription start site CpG content in bovine.** Histogram of gene counts with 0–16% CpG content in 1000 bp surrounding transcription start sites in the bovine genome (bosTau4).
(TIF)
