# Supplementary figures and images for: Genome-Wide DNA Methylation Patterns and Transcription Analysis in Sheep Muscle
Source: PLoS One. 2014 Jul 10;9(7):e101853. doi: 10.1371/journal.pone.0101853 (PMC4092064; doi:10.1371/journal.pone.0101853)

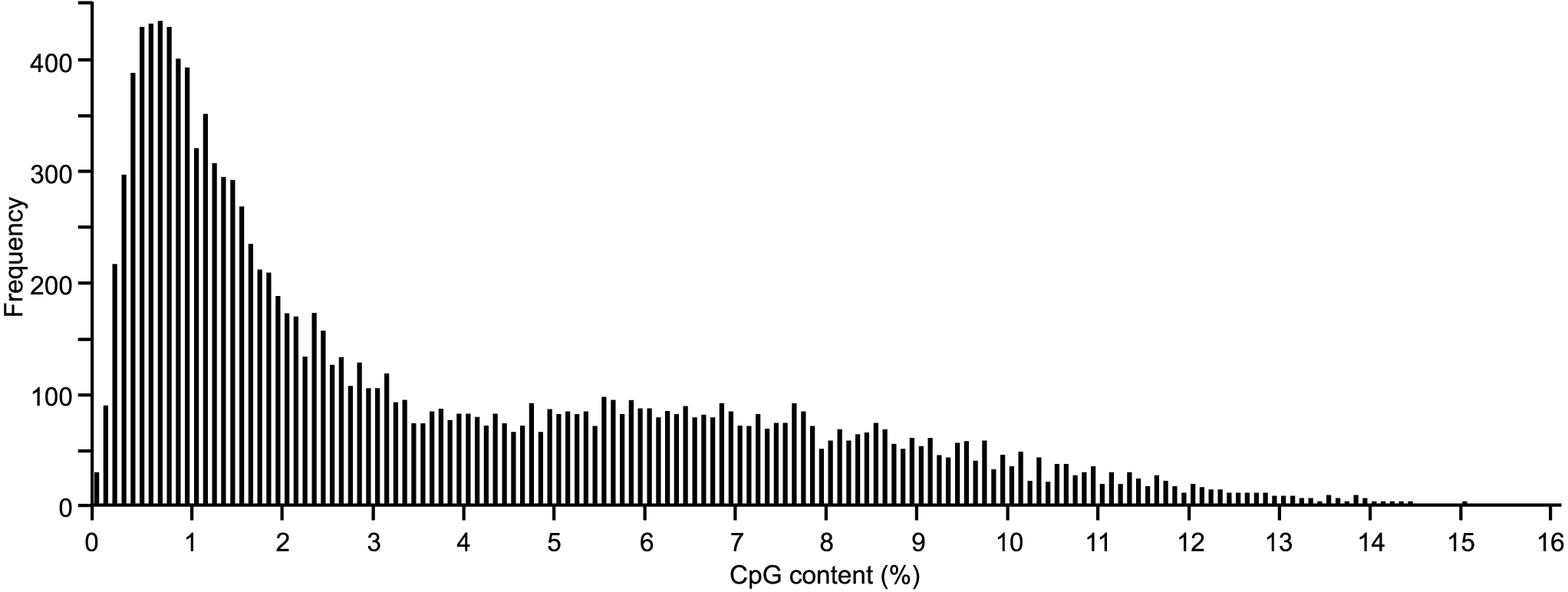

Supplement: Figure S1 — Transcription start site CpG content in bovine. Histogram of gene counts with 0–16% CpG content in 1000 bp surrounding transcription start sites in the bovine genome (bosTau4). (TIF) [file pone.0101853.s001.tif]
